# Parental mental health, socioeconomic position and the risk of asthma in children—a nationwide Danish register study

**DOI:** 10.1093/eurpub/ckab205

**Published:** 2021-12-10

**Authors:** Signe Heuckendorff, Martin Nygård Johansen, Charlotte Overgaard, Søren Paaske Johnsen, Yvonne Kelly, Kirsten Fonager

**Affiliations:** 1 Department of Social Medicine, Aalborg University Hospital, Aalborg, Denmark; 2 Department of Clinical Medicine, Danish Center for Clinical Health Services Research (DACS), Aalborg University, Aalborg, Denmark; 3 Unit of Clinical Biostatistics, Aalborg University Hospital, Aalborg, Denmark; 4 Public Health and Epidemiology Group, Department of Health Science and Technology, Aalborg University, Aalborg, Denmark; 5 Department of Clinical Medicine, Aalborg University, Aalborg, Denmark; 6 Research Department of Epidemiology and Public Health, University College London, London, UK

## Abstract

**Background:**

Parental mental illness affects child health. However, less is known about the impact of different severities of maternal depression and anxiety as well as other mental health conditions. The objective of this study was to examine the impact of different severities of maternal and paternal mental health conditions on child asthma.

**Methods:**

This nationwide, register-based cohort study included all children in Denmark born from 2000 to 2014. Exposure was parental mental health conditions categorized in three severities: minor (treated at primary care settings), moderate (all ICD-10 F-diagnoses given at psychiatric hospital) and severe (diagnoses of severe mental illness). The children were followed from their third to sixth birthday. Child asthma was identified by prescribed medication and hospital-based diagnoses. Incidence rate ratios were calculated using negative binomial regression analyses.

**Results:**

The analyses included 925 288 children; 26% of the mothers and 16% of the fathers were classified with a mental health condition. Exposed children were more likely to have asthma (10.6–12.0%) compared with unexposed children (8.5–9.0%). The three severities of mental health conditions of the mother and the father increased the risk of child asthma, most evident for maternal exposure. Additive interaction between maternal mental health conditions and disadvantaged socioeconomic position was found.

**Conclusion:**

We found an increased risk of asthma in exposed children, highest for maternal exposure. Not only moderate and severe, but also minor mental health conditions increased the risk of child asthma. The combination of mental health condition and disadvantaged socioeconomic position for mothers revealed a relative excess risk.

## Introduction

A high number of children grow up in families with parental mental illness. Studies from the UK,^1^ Sweden^2^ and Denmark^3^ report that one in 5–10 children have a parent with mental illness and may be at increased risk of health disadvantages, e.g. asthma, obesity and injuries.^4^

Asthma is one of the major childhood morbidities affecting 5–10% of children.^5^ Poor parental mental health is argued to negatively influence child health, including asthma.

Previous studies found that chronic (more periods of exposure) maternal distress (depression or anxiety) before childbirth and during the child’s first years of life, rather than a critical period (only one period of exposure), was associated with increased risk of offspring asthma.^6^ However, the studies examining parental mental health and child asthma primarily illuminates the risks for offspring following depression and anxiety among the mothers.^4^^,^^6^^,^^7^ The severity of the mental health condition rather than the diagnosis itself might impact the risk of child asthma.^8^ Furthermore, although fathers are becoming more engaged as caregivers,^9^ the literature on fathers mental health and child morbidity is sparse.^4^

Parental mental health conditions and socioeconomic position are both described as social determinants of child health and potential pathways leading to child health inequalities.^10^ The relationship between mental health and socioeconomic position is bidirectional: poor mental health lead to reduced income and employment inducing poverty and in turn increases the risk of poor mental health.^11^ Furthermore, people in socioeconomic disadvantage suffer disproportionately from poor mental health and the consequence hereof.^11^ Poor parental mental health may therefore interact with socioeconomic position, potentially yielding an even higher risk of child morbidity.

Studies argue that disadvantaged socioeconomic position is related to poor mental health and adjust for socioeconomic factors.^4^^,^^12^ However, to the best of our knowledge, no prior studies have examined whether these factors interact to describe a potential excess risk for children of parents with the combination of mental health conditions and disadvantaged socioeconomic position.

We aimed to address the described knowledge gaps by examining the impact of different severities of mental health conditions of the mother as well as the father on child asthma. Furthermore, we examined whether the impact of mental health conditions interacted with disadvantaged socioeconomic position.

## Methods

### Study design and data sources

We conducted a register-based cohort study using nationwide registers. Data linkages were achieved via the personal identity number, which is assigned to all Danish residents at birth or upon taking up residency.^13^ Register keepers at Statistics Denmark carried out data collection and register linkage with all data having been anonymized before the researchers gained access to the data.

The following registers were used:

- The National Patient Register,^14^ which contains information about diagnoses, admissions and outpatient contacts. The diagnoses were encoded using the International Classification of Diseases, 10th Revision (ICD-10).
- The Danish Health Service Register for Primary Care^15^ provided information on contacts to general practitioners (GPs), private psychiatrists and psychologists.
- The Danish National Prescription Registry,^16^ which contains information on reimbursed drug prescriptions and uses the Anatomical Therapeutic Chemical (ATC) Classification System.
- The Population Education Registry,^17^ which contains information on parental highest completed education.
- The Income Statistics Register,^18^ which contains information on socioeconomic classification.
- The Danish Civil Registration System,^13^ which contains date of birth, parental cohabitation status and child gender.

The included Danish registers are in general considered to be of high quality with complete long-term follow-up.^19^

Ethical approval is not needed for register-based studies in Denmark. The project was approved by Statistics Denmark before gaining access to the data.

### Settings, study population and follow-up

The Danish healthcare system is characterized by free access. Services from general practitioner (GP) and other specialities, such as psychiatry, practising outside hospitals as well as from public hospitals are funded by the Danish tax system and free of charge.

All children in Denmark born from 1 January 2000 to 31 December 2014 were identified. Baseline was defined at the time of the child turning 3 years old. The children were followed until their sixth birthday, death, emigration from Denmark or 15 March 2018, whichever came first. Asthma cases were defined between the age of three and six because onset of asthma for the majority is at preschool.^20^ Children younger than 3 years were not included as lower respiratory tract infections in the first 3 years of life are common, probably due to smaller airways, and the children do not have increased risk of asthma.^21^ Thus prescriptions of asthma medication before the age of three are more likely due to lower respiratory tract infections rather than asthma. The exposure period of parental mental health was 5 years before baseline; i.e. 2 years prior to birth until the third birthday of the child. This exposure was chosen as a registered mental health condition at a given time-point reflects a mental health condition during a broader time period. A Danish survey found that three-fourth of the parents reporting a mental illness have had the mental illness for more than 5 years.^3^

**Figure 1:**
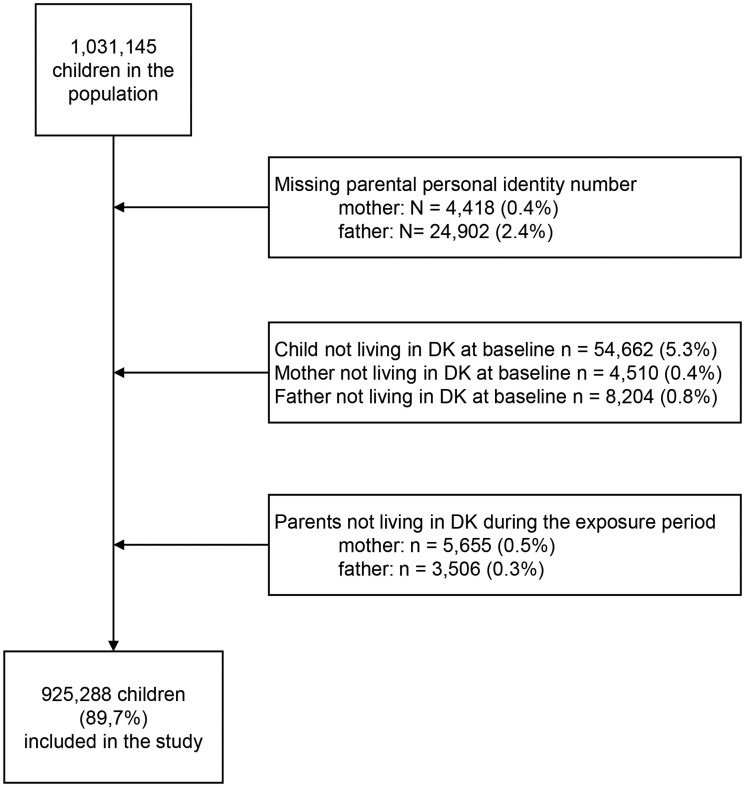
Flow chart of the study population

Children were excluded if they or their parents did not live in Denmark at baseline, if the parents did not live in Denmark during the exposure period or if the parental personal identity number was missing (figure 1).

### Parental mental health

Parental mental health was categorized in four mutually exclusive groups: no mental health condition, minor, moderate and severe. The Danish Health Service Register for Primary Care provide information from the primary health sector where minor mental health conditions in Denmark are treated by GPs and psychologists. However, this register does not contain information on diagnoses; therefore, minor mental health conditions needed to be identified otherwise. Treatments such as talk therapy and psychometric tests performed by GPs as well as contacts to psychologists are registered and were used. Furthermore, prescriptions of antidepressants and anxiolytics were used.

The criteria for minor mental health conditions were:

- At least two prescriptions of antidepressant medication (ATC N06AB, N06AX) *and/or*
- At least two prescriptions of anxiolytics (benzodiazepines: ATC N03AE, N05BA, N05CD, N05CF) *and/or*
- At least two sessions of talk therapy by general practitioner *and/or*
- At least two psychometric tests at general practice *and/or*
- At least one contact to private psychologist
- And no contacts to psychiatrist (neither outside psychiatric hospital nor at psychiatric hospital)

The criteria for moderate mental health conditions were:

- Mental health conditions treated at psychiatrists outside psychiatric hospital (including child and adolescent psychiatrists) *and/or*
- Any registered psychiatric diagnosis (ICD-10 F00-99) at psychiatric hospital except diagnoses included in the severe group

The criteria for severe mental health conditions were an ICD-10 diagnosis of the below-mentioned registered at psychiatric hospital:

- F20-22: Schizophrenia *and/or*
- F30-31: Bipolar disease *and/or*
- F32-34: Unipolar depression if also having a registered psychiatric hospital admission *and/or*
- F60.3: Emotionally unstable personality disorder if also having a registered psychiatric hospital admission

The reference group were defined as none of the criteria of minor, moderate or severe mental health conditions fulfilled.

### Socioeconomic position

To examine the interaction between mental health and socioeconomic position, a dichotomous variable was created based on the main source of income or employment at baseline.^18^ The disadvantaged group was defined as having no affiliation to the labour market or education (disability pension, cash benefits or similar).

### Child asthma

We defined an outcome of asthma as:

- At least two prescriptions of either inhaled long-acting β_2_-agonist (ATC R03AC), fixed-dose combinations of inhaled β_2_-agonists and corticosteroids (ATC R03AK), inhaled corticosteroids (ATC R03BA) or leukotriene-receptor antagonist (ATC R03DC) and/or
- A hospital diagnosis of asthma (ICD-10 J45) or status asthmaticus (ICD-10 J46)

### Covariates

Covariates that could potentially confound the association between parental mental health conditions and child asthma were identified by reviewing the literature. A directed acyclic graph (DAG) was constructed to evaluate the identified covariates (see Supplementary data).^22^ To account for the changes in use of mental health services and medications during the study period, we included calendar year as a covariate. All covariates were extracted at baseline.

Parental education was based on the International Standard Classification of Education (ISCED 2011)^23^ and grouped according to highest completed education into three categories [12 years or below (ISCED level 0–2), 12–14 years (ISCED level 3–4) or above 14 years (ISCED level 5–8)].^23^ Parental cohabitation status was dichotomized in single or cohabitant. We adjusted for parental age at the time of birth of the child and calendar year using restricted cubic splines with three knots.^24^ Parental asthma was defined as a history of a hospital diagnosis of asthma (ICD-10 J45-46).

### Statistics

Analyses were done separately for the mother and the father.

At baseline, the total number of children and percentages of all covariates were calculated. For each outcome, number and percentages of children with at least one outcome event during the follow-up period in each exposure group were calculated. Negative binomial regression was chosen due to overdispersion in the data. Incidence rate ratios (IRRs) for the rate of the outcome events were calculated of the event counts comparing the three exposure groups to the reference group. To account for siblings (with the same mother and father), the estimates were fitted using generalized estimating equations with an exchangeable correlation structure. The adjusted analyses are based on complete case data.

Subgroup analyses within each level of mental health condition were performed; for minor mental health conditions based on mental health services and prescriptions of medication, for moderate and severe mental health conditions based on diagnoses.

To examine a potential interaction between mental health condition and socioeconomic position, the relative excess risk due to interaction (RERI) was calculated within each stratum of parental mental health condition to describe the additive interaction between mental health (none, minor, moderate, severe) and socioeconomic position (SEP0, SEP1),^25^ such as:

$${\mathrm{RERI}}_{\mathrm{minor}}=\frac{{\mathrm{IRR}}_{\mathrm{minor},\mathrm{SEP}1}-{\mathrm{IRR}}_{\mathrm{minor},\mathrm{SEP}0}-{\mathrm{IRR}}_{\mathrm{none},\mathrm{SEP}1}+{\mathrm{IRR}}_{\mathrm{none},\mathrm{SEP}0}}{{\mathrm{IRR}}_{\mathrm{none},\mathrm{SEP}0}}$$

A sensitivity analysis was performed to examine whether chronic conditions in early childhood, e.g. chromosome anomalies and birth defects, had an impact on child asthma excluding children with a diagnosis prior to baseline (full list of diagnoses in Supplementary data). Furthermore, an analysis with the outcome separate as either asthma medication or asthma diagnosis was performed.

## Results

We identified 1 031 145 children. Due to missing parental information or if the child did not live in the country at baseline, 10% of the children were excluded (figure 1). A total of 925 288 (89.7%) children were included in the analyses (table 1); 26% of the mothers and 16% of the fathers were classified with a mental health condition. If the mother was classified with no mental health condition, 10.7% were living in disadvantaged circumstances. This was the case for a higher share, 12.5% if the mother had a minor mental health condition, 30.1% if having a moderate and 46.8% if having a severe mental health condition. For the father, 4.6%, 11.3%, 30.1% and 50.7% were living in disadvantaged circumstances if they were classified with no, minor, moderate and severe mental health condition, respectively.

**Table 1:** Baseline, percentages of socioeconomic characteristics and parental asthma by group of parental mental health condition

	Mother mental health condition				Father mental health condition			
	None	Minor	Moderate	Severe	None	Minor	Moderate	Severe
Children, *N* (%)	688 025 (74.4)	159 702 (17.3)	69 486 (7.5)	8075 (0.9)	775 400 (83.8)	98 650 (10.7)	44 095 (4.8)	7143 (0.8)
Parental age at birth of the child								
<25	11.0	12.5	22.8	26.1	5.3	6.4	13.0	13.7
25–32	49.9	46.8	43.9	43.5	38.6	34.3	35.6	34.9
>32	39.0	40.7	33.4	30.3	56.0	59.3	51.4	51.4
Missing	0.1	0.0	0.0	0.1	0.2	0.0	0.0	0.0
SEP								
Affiliated to labour marked/education	88.7	87.5	69.9	53.0	94.9	88.7	69.9	49.2
Disadvantaged: no affiliation to labour marked/education	10.7	12.5	30.1	46.8	4.6	11.3	30.1	50.7
Missing	0.6	0.0	0.0	0.2	0.5	0.0	0.1	0.1
Educational level								
Short/ISCED 0–2	14.9	17.6	33.9	42.0	16.1	22.1	38.2	44.7
Medium/ISCED 3–4	36.8	37.7	36.2	34.3	45.4	43.7	38.8	35.1
Long/ISCED 5–8	46.6	43.9	28.6	22.3	36.8	32.4	19.7	15.6
Missing	1.7	0.8	1.3	1.5	1.7	1.7	3.3	4.5
Country of origin								
Nordic	84.5	90.4	86.4	86.5	86.3	87.0	77.5	72.2
Western Europe	1.4	1.0	0.8	1.0	1.9	1.6	1.4	1.5
Other	13.3	8.5	12.6	12.2	11.0	11.2	20.8	26.0
Missing	0.8	0.1	0.2	0.3	0.8	0.2	0.3	0.3
Cohabitation status								
Single	6.5	13.9	23.2	32.2	6.8	15.5	26.7	37.7
Cohabitant	93.3	86.1	76.7	67.6	92.9	84.4	73.1	62.1
Missing	0.2	0.0	0.1	0.1	0.3	0.1	0.1	0.2
Diagnosis of asthma								
Yes	2.8	4.8	6.6	7.6	2.1	3.3	4.7	4.9

**Figure 2:**
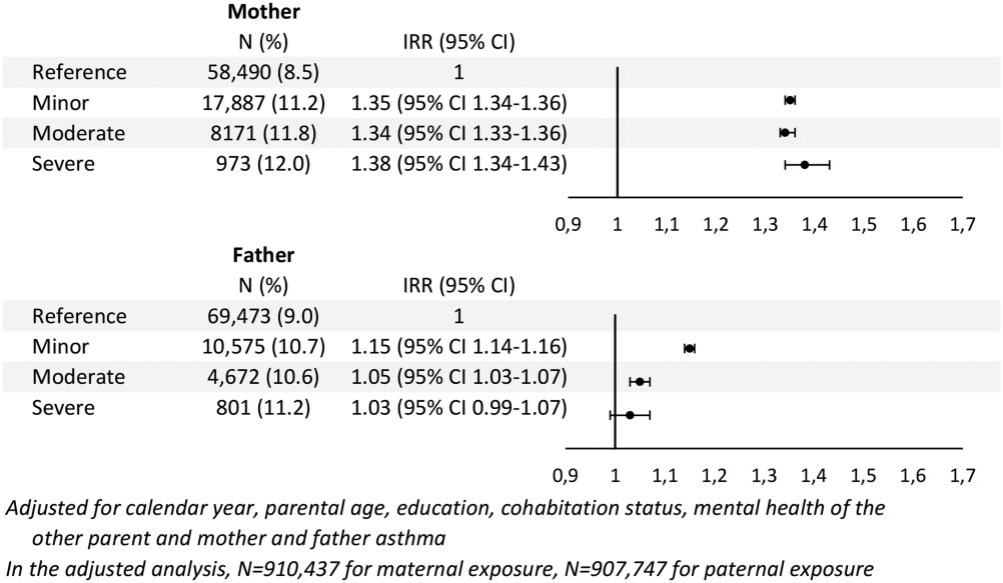
Adjusted incidence rate ratios (IRR) for the rate of asthma events based on maternal and paternal mental health condition

Overall, children of parents with poor mental health were more likely to have asthma (figure 2). In the reference groups (children of parents with no mental health condition), 8.5% and 9.0% of the children were found with at least one event asthma, whereas this was the case for 10.7–12.0% in the exposed groups.

Adjusted analyses (figure 2) revealed significant higher IRRs for asthma in children of parents with a mental health condition compared with children of parents without a mental health condition. The only exceptions were severe mental health condition of the father where the estimates were non-significant. Overall, the impact of mental health condition on child asthma was stronger for mothers than fathers. The association between maternal minor, moderate and severe mental health condition and child asthma were similar [minor: IRR: 1.35 (95% CI: 1.34–1.36); moderate: IRR: 1.34 (95% CI: 1.33–1.36) and severe: IRR: 1.38 (95% CI: 1.34–1.43)].

Supplementary eTable S1 shows the IRRs of subgroups parental mental health conditions on child asthma. Overall, the estimates were similar (Supplementary data).

The interaction analyses (table 2) for asthma showed additive interaction between disadvantaged maternal socioeconomic position and minor [RERI: 0.07 (95% CI: 0.04–0.11)], moderate [RERI: 0.08 (95% CI: 0.05–0.12)] and especially severe mental health condition [RERI: 0.21 (95% CI: 0.12–0.30)]. The analyses indicated no additive interaction between disadvantaged socioeconomic position and mental health condition of the father.

**Table 2:** Relative excess risk (RERI) on child asthma due to interaction between parental mental health conditions (minor, moderate, severe) and disadvantaged socioeconomic position

Asthma, RERI (95% CI)				
	Crude		Adjusted^a^	
	Mother	Father	Mother	Father
Minor	0.14 (95% CI: 0.10–0.17)	–0.05 (95% CI: –0.09 to –0.01)	0.07 (95% CI: 0.04–0.11)	–0.06 (95% CI: –0.10 to –0.03)
Moderate	0.13 (95% CI: 0.10–0.17)	–0.05 (95% CI: –0.09 to –0.01)	0.08 (95% CI: 0.05–0.12)	–0.04 (95% CI; –0.08 to –0.01)
Severe	0.31 (95% CI: 0.21–0.10)	0.06 (95% CI: –0.02 to 0.15)	0.21 (95% CI: 0.12–0.30)	0.13 (95% CI: 0.06–0.21)

a Adjusted for calendar year, parental age, education, cohabitation, mental health of the other parent and mother and father asthma.

Sensitivity analysis excluding children with prior diagnoses did not change the estimates noteworthy (Supplementary eTable S2). Analysis of the outcomes separated by asthma medication and diagnosis yielded overall similar results (Supplementary eTable S3).

## Discussion

### Main findings

We report increased rates of asthma for children whose parents have poor mental health, and this was evident for both maternal and paternal exposure. However, only minor association for paternal exposure was found. Not only moderate and severe, but also minor mental health conditions were associated with increased rate of child asthma. An additive interaction of poor maternal mental health and disadvantaged socioeconomic position was found on child asthma.

### Strengths of the study

Data from high-quality nationwide registries with few missing data on an entire birth cohort provided powerful statistical analyses. We used strict definitions of exposure and outcome to avoid reverse causation bias. The Danish registries are considered valid in terms of completeness, registration processes and accuracy.^19^ The mental health conditions as well as child asthma were based on health professional assessments unlike several previous studies.^26–29^ Furthermore, we were able to obtain exposure information of the fathers unlike previous studies.^6^^,^^26^^,^^30^^,^^31^

### Limitations of the data

There is a risk of misclassification of minor parental mental health condition as we cannot access diagnoses, indication of prescribed medication or talk therapy or the results of the psychometric tests from The Danish Health Service Register for Primary Care. Furthermore, we only identified the parents with poor mental health who sought medical care. Thus, parents with poor mental health without any mental health condition-related contacts to GP, private psychologist, prescribed antidepressants or anxiolytic drugs or contacts to psychiatrist or psychiatric hospital were misclassified as being in the reference group. Moreover, parents with psychiatric disorder who have no records at psychiatric hospital within 5 years before baseline were also misclassified as being in the reference group. Likewise, we were not able to identify children with asthma without prescriptions or hospital diagnoses. These misclassifications may underestimate the association between parental mental health conditions and child asthma.

Since we did not have information on the asthma phenotype, the interpretation of the associations between parental mental health conditions and child asthma should be in the light of the complexity of instrumenting asthma in routine health data.

We did not have information on factors like housing conditions and parental smoking status, which might impact child asthma. Especially smoking might be a significant mediator; thus, some of the association between parental mental health conditions and offspring asthma may be due to smoking. However, these factors are closely related to the covariates included in our analyses.^32^ Furthermore, the DAG may involve limitations as we did not have information of the timing of the included covariates.

### Interpretation

Our results support previous research linking maternal anxiety and depression and child asthma,^4^ severe parental mental illness and child morbidity including asthma.^12^ To the best of our knowledge, this is the first study using a large, nationwide cohort including information from the primary health sector to examine the association between a different severities of parental mental health conditions and the association with child asthma.

The overall effects of the different subgroups within each level of mental health conditions were similar (Supplementary eTable S1), and shared mechanisms might explain at least some of the association between parental mental health conditions and child asthma such as physiological components and health behaviours.

Socioeconomic disadvantage as well as poor parental mental health are often classified as early life stressors,^33^^,^^34^ and might share several mechanisms when explaining the association with increased risk of child asthma. Perinatal stress and early life stress seem to influence respiratory processes and lung function through disruptive neuroendocrine function and imbalance of the autonomic nervous system.^35^ Due to developmental plasticity of the respiratory system, these exposures might lead to both transient and more long-term effects.^35^

Health behaviour is another mechanism explaining some of the association between both poor parental mental health and socioeconomic disadvantages and child morbidity.^10^ Poor mental health may interfere with parents’ capacity to communicate their children’s need to healthcare professionals and follow general health advice. Moreover, poor mental health and socioeconomic disadvantages are associated with increased risk of smoking,^36^ leading to an increased risk of child asthma.^37^ Furthermore, children of parents with poor mental health may have more contacts to hospitals and GPs,^38^ and parents with poor mental health might be more likely to seek help to minor health problems in their children.

The association between mental health conditions of the father and child asthma was overall sparse and strongest for minor mental health condition, which might lead to the interpretation that the association could be due to residual confounding as also reported by Brew et al.^6^

The only sparse association with paternal exposure and absence of additive interaction might be explained by the influence of environmental factors such as intrauterine negative effects on the foetus and maternal lifestyle factors associated with mental health conditions. Moreover, children of parents with severe mental illness are more likely to co-habit with their mother than father,^39^ thus depending most upon mother’s care.

The Danish welfare state is characterized by free access to universal healthcare and social benefits. However, we still find, in line with a Swedish study,^2^ a markedly larger share of socioeconomic disadvantaged in children of parents with mental health conditions. Furthermore, we found an additional increased risk for these children with double adverse exposure, which should call for public health attention. Interventions focusing on socioeconomic adversity should also consider the barriers of poor mental health.^2^ Currently, supporting services are targeted the parents with poor mental health and not the children.^40^ These children could however easily be identified through their parents’ contacts with psychiatric service or primary healthcare.^40^ It is notable that all severities of maternal mental health conditions, including minor conditions treated only at primary healthcare, overall yielded similar effect on child asthma. The findings highlight the importance of a broader focus of parental mental health and not only severe mental illness. The mechanisms behind the described associations need to be explored before targeted interventions can be implemented.

The results are generalizable to Danish setting but may be problematic outside Denmark as the healthcare-seeking behaviour probably is related to the structure of the Danish healthcare system. However, the overall findings of associations between parental mental health and child health has broad applicability.

## Conclusions

This nationwide study examined morbidity in children exposed to parental mental health conditions. We found an increased risk of asthma in exposed children, highest for maternal exposure. We showed that not only moderate and severe, but also minor mental health conditions increased the risk of child asthma. Moreover, an additional risk in children of mothers with the combination of mental health condition and disadvantaged socioeconomic position was revealed. Our results highlight a public health concern, and detection of the children at risk is essential.

## Supplementary data

Supplementary data are available at *EURPUB* online. 

## Funding

This work was supported by Helsefonden (grant number: 19-B-0093). Helsefonden did not influence the design or conduct of the study or its management, analysis and interpretation of the data, and neither did it review or approve the manuscript before submission. 

*Conflicts of interest*: None declared.

Key points

- Parental mental health conditions, even minor mental health conditions, were associated with increased offspring risk of asthma.
- The risk was most evident for maternal mental health conditions.
- The combinations of disadvantaged socioeconomic position and mental health conditions of the mother showed additive interaction for child preschool asthma.

## Supplementary Material

ckab205_Supplementary_Data

## References

[1] Abel KM , Hope H, Swift E, et al Prevalence of maternal mental illness among children and adolescents in the UK between 2005 and 2017: a national retrospective cohort analysis. Lancet Public Health 2019;4:e291–300. 31155222 10.1016/S2468-2667(19)30059-3 PMC6557735

[2] Pierce M , Abel KM, Muwonge J, et al Prevalence of parental mental illness and association with socioeconomic adversity among children in Sweden between 2006 and 2016: a population-based cohort study. Lancet Public Health 2020;5:e583–91. 33120044 10.1016/S2468-2667(20)30202-4

[3] Christesen AMS, Knudsen CK, Fonager K, et al. Prevalence of parental mental health conditions among children aged 0–16 years in Denmark: A nationwide register-based cross-sectional study. Scand J Public Health 2021;140349482110454. 10.1177/14034948211045462 34609273

[4] Pierce M , Hope HF, Kolade A, et al Effects of parental mental illness on children’s physical health: systematic review and meta-analysis. Br J Psychiatry 2020;217:354–63. 31610824 10.1192/bjp.2019.216

[5] Sennhauser FH , Braun-Fahrländer C, Wildhaber JH. The burden of asthma in children: a European perspective. Paediatr Respir Rev 2005;6:2–7. 15698807 10.1016/j.prrv.2004.11.001

[6] Brew BK , Lundholm C, Viktorin A, et al Longitudinal depression or anxiety in mothers and offspring asthma: a Swedish population-based study. Int J Epidemiol 2018;47:166–74. 29040553 10.1093/ije/dyx208 PMC5837783

[7] Alton ME , Zeng Y, Tough SC, et al Postpartum depression, a direct and mediating risk factor for preschool wheeze in girls. Pediatr Pulmonol 2016;51:349–57. 26448278 10.1002/ppul.23308

[8] Reupert AE , Maybery DJ, Kowalenko NM. Children whose parents have a mental illness: prevalence, need and treatment. Med J Aust 2013;199:S7–9. 10.5694/mja11.11200 25369850

[9] van der Gaag N , Heilman B, Gupta T, et al State of the World’s Fathers: Unlocking the Power of Men’s Care. Washington, DC: Promundo-US, 2019.

[10] Pearce A , Dundas R, Whitehead M, Taylor-Robinson D. Pathways to inequalities in child health. Arch Dis Child 2019;104:998–1003. 30798258 10.1136/archdischild-2018-314808 PMC6889761

[11] World Health Organization, Calouste Gulbenkian Foundation. Social Determinants of Mental Health. Geneva: World Health Organization, 2014. doi:10.1007/978-3-319-59123-0_4.

[12] Ranning A , Benros ME, Thorup AAE, et al Morbidity and mortality in the children and young adult offspring of parents with schizophrenia or affective disorders—a nationwide register-based cohort study in 2 million individuals. Schizophr Bull 2020;46:130–9. 31173637 10.1093/schbul/sbz040 PMC6942150

[13] Pedersen CB. The Danish civil registration system. Scand J Public Health 2011;39:22–5. 21775345 10.1177/1403494810387965

[14] Schmidt M , Schmidt SAJ, Sandegaard JL, et al The Danish National Patient Registry: a review of content, data quality, and research potential. Clin Epidemiol 2015;7:449–90. 26604824 10.2147/CLEP.S91125 PMC4655913

[15] Sahl Andersen J , De Fine Olivarius N, Krasnik A. The Danish National Health Service Register. Scand J Public Health 2011;39:34–7. 21775348 10.1177/1403494810394718

[16] Wallach Kildemoes H , Toft Sørensen H, Hallas J. The Danish National Prescription Registry. Scand J Public Health 2011;39:38–41. 21775349 10.1177/1403494810394717

[17] Jensen VM , Rasmussen AW. Danish education registers. Scand J Public Health 2011;39:91–4. 21775362 10.1177/1403494810394715

[18] Baadsgaard M , Quitzau J. Danish registers on personal income and transfer payments. Scand J Public Health 2011;39:103–5. 21775365 10.1177/1403494811405098

[19] Schmidt M , Schmidt SAJ, Adelborg K, et al The Danish health care system and epidemiological research: from health care contacts to database records. Clin Epidemiol 2019;11:563–91. 31372058 10.2147/CLEP.S179083 PMC6634267

[20] Fuchs O , Bahmer T, Rabe KF, von Mutius E. Asthma transition from childhood into adulthood. Lancet Respir Med 2017;5:224–34. 27666650 10.1016/S2213-2600(16)30187-4

[21] Martinez FD , Wright AL, Taussig LM, et al Asthma and wheezing in the first six years of life. N Engl J Med 1995;332:133–8. 7800004 10.1056/NEJM199501193320301

[22] Greenland S , Pearl J, Robins JM. Causal diagrams for epidemiologic research. Epidemiology 1999;10:37–48. 9888278

[23] United Nation Educational Scientific and Cultural Organisation (UNESCO). International Standard Classification of Education, 2011. Available at: http://uis.unesco.org/sites/default/files/documents/international-standard-classification-of-education-isced-2011-en.pdf (24 February 2021, date last accessed). 29058848

[24] Desquilbet L , Mariotti F. Dose-response analyses using restricted cubic spline functions in public health research. Stat Med 2010;29:1037–57. 20087875 10.1002/sim.3841

[25] Jackson JW , Williams DR, VanderWeele TJ. Disparities at the intersection of marginalized groups. Soc Psychiatry Psychiatr Epidemiol 2016;51:1349–59. 27531592 10.1007/s00127-016-1276-6 PMC5350011

[26] Kozyrskyj AL , Letourneau NL, Kang LJ, Salmani M. Associations between postpartum depressive symptoms and childhood asthma diminish with child age. Clin Exp Allergy 2017;47:324–30. 27770463 10.1111/cea.12837

[27] Schuez-Havupalo L , Lahti E, Junttila N, et al Parents’ depression and loneliness during pregnancy and respiratory infections in the offspring: a prospective birth cohort study. PLoS One 2018;13:e0203650. 30192872 10.1371/journal.pone.0203650 PMC6128609

[28] van de Loo KFE , van Gelder MMHJ, Roukema J, et al Prenatal maternal psychological stress and childhood asthma and wheezing: a meta-analysis. Eur Respir J 2016;47:133–46. 26541526 10.1183/13993003.00299-2015

[29] Magnus MC , Wright RJ, Røysamb E, et al Association of maternal psychosocial stress with increased risk of asthma development in offspring. Am J Epidemiol 2018;187:1199–209. 29244063 10.1093/aje/kwx366 PMC5982733

[30] Khashan AS , Wicks S, Dalman C, et al Prenatal stress and risk of asthma hospitalization in the offspring: a Swedish population-based study. Psychosom Med 2012;74:635–41. 22753636 10.1097/PSY.0b013e31825ac5e7

[31] Kozyrskyj AL , Mai X-M, McGrath P, et al Continued exposure to maternal distress in early life is associated with an increased risk of childhood asthma. Am J Respir Crit Care Med 2008;177:142–7. 17932381 10.1164/rccm.200703-381OC

[32] Casetta B , Videla AJ, Bardach A, et al Association between cigarette smoking prevalence and income level: a systematic review and meta-analysis. Nictine Tob Res 2017;19:1401–7. 10.1093/ntr/ntw266 27679607

[33] Fagundes CP , Glaser R, Kiecolt-Glaser JK. Stressful early life experiences and immune dysregulation across the lifespan. Brain Behav Immun 2013;27:8–12. 22771426 10.1016/j.bbi.2012.06.014 PMC3518756

[34] Oh DL , Jerman P, Silvério Marques S, et al Systematic review of pediatric health outcomes associated with childhood adversity. BMC Pediatr 2018;18:83. 29475430 10.1186/s12887-018-1037-7 PMC5824569

[35] Wright RJ. Perinatal stress and early life programming of lung structure and function. Biol Psychol 2010;84:46–56. 20080145 10.1016/j.biopsycho.2010.01.007 PMC2888999

[36] Orton S , Jones LL, Cooper S, et al Predictors of children’s secondhand smoke exposure at home: a systematic review and narrative synthesis of the evidence. PLoS One 2014;9:e112690. 25397875 10.1371/journal.pone.0112690 PMC4232519

[37] Burke H , Leonardi-Bee J, Hashim A, et al Prenatal and passive smoke exposure and incidence of asthma and wheeze: systematic review and meta-analysis. Pediatrics 2012;129:735–44. 22430451 10.1542/peds.2011-2196

[38] Heuckendorff S , Johansen MN, Johnsen SP, et al Parental mental health conditions and use of healthcare services in children the first year of life—a register-based, nationwide study. BMC Public Health 2021;21:557. 33743653 10.1186/s12889-021-10625-y PMC7981963

[39] Ranning A , Munk Laursen T, Thorup A, et al Children of parents with serious mental illness: with whom do they grow up? A prospective, population-based study. J Am Acad Child Adolesc Psychiatry 2016;55:953–61. 27806863 10.1016/j.jaac.2016.07.776

[40] Abel KM , Hope H, Faulds A, Pierce M. Promoting resilience in children and adolescents living with parental mental illness (CAPRI): children are key to identifying solutions. Br J Psychiatry 2019;215:513–5. 10.1192/bjp.2019.118 31190644

