# Supplementary material for: Parental mental health, socioeconomic position and the risk of asthma in children—a nationwide Danish register study
Source: Eur J Public Health. 2021 Dec 10;32(1):14–20. doi: 10.1093/eurpub/ckab205 (PMC8807069; doi:10.1093/eurpub/ckab205)
Supplement: ckab205_Supplementary_Data [file ckab205_supplementary_data.zip › ejph-2021-02-om-0244-File005.docx]

**Supplements**

**eTable 1. Adjusted incidence rate ratios (IRR) and 95% confidence intervals (95% CI) of asthma based on maternal and paternal mental health in subgroups of each level of mental health condition**

*Adjusted for parental age, education, cohabitation status, calendar year, mental health of the other parent and mother and father asthma*

| **Maternal mental health conditions** | |
| --- | --- |
| Minor group overall | 1.35 (95% CI 1.34-1.36) |
| **Minor subgroups** | |
| Antidepressants | 1.38 (95% CI 1.36-1.41) |
| Benzodiazepines | 1.57 (95% CI 1.54-1.61) |
| Psychologist | 1.43 (95% CI 1.41-1.45) |
| Psychometric test at GP | 1.34 (95% CI 1.32-1.36) |
| Supportive conversation/talk therapy at GP | 1.33 (95% CI 1.31-1.34) |
|  |  |
| Moderate group overall | 1.34 (95% CI 1.33-1.36) |
| **Moderate subgroups** | |
| F10-19, Mental and behavioural disorders due to psychoactive substance use | 1.39 (95% CI 1.35-1.44) |
| F32-34, Unipolar depression | 1.32 (95% CI 1.29-1.36) |
| F40-41, Anxiety disorders | 1.36 (95% CI 1.32-1.40) |
| F42-49, Other anxiety and stress-related disorders | 1.35 (95% CI 1.32-1.38) |
| F60-62, Personality disorders | 1.47 (95% CI 1.42-1.52) |
| F00-09,35-39,50-59,63-99, Other F-diagnoses | 1.47 (95% CI 1.43-1.52) |
| At least one contact to private psychiatrist | 1.35 (95% CI 1.32-1.37) |
|  |  |
| Severe group overall | 1.38 (95% CI 1.34-1.43) |
| **Severe subgroups** | |
| F20-29 Schizophrenia and psychoses | 1.41 (95% CI 1.34-1.50) |
| F30-31 Bipolar | 1.28 (95% CI 1.17-1.39) |
| F32-34 Unipolar depression with admission | 1.32 (95% CI 1.27-1.38) |
| F60.3 Borderline personality disorder with admission | 1.60 (95% CI 1.49-1.72) |
|  |  |
| **Paternal mental health conditions** | |
| Minor group overall | 1.14 (95% CI 1.13-1.15) |
| **Minor subgroups** | |
| Antidepressants | 1.21 (95% CI 1.19-1.23) |
| Benzodiazepines | 1.08 (95% CI 1.06-1.11) |
| Psychologist | 1.22 (95% CI 1.20-1.25) |
| Psychometric test at GP | 1.20 (95% CI 1.17-1.22) |
| Supportive conversation/talk therapy at GP | 1.11 (95% CI 1.09-1.12) |
|  |  |
| Moderate group overall | 1.03 (95% CI 1.01-1.04) |
| **Moderate subgroups** | |
| F10-19, Mental and behavioural disorders due to psychoactive substance use | 1.03 (95% CI 0.99-1.06) |
| F32-34, Unipolar depression | 1.00 (95% CI 0.95-1.05) |
| F40-41, Anxiety disorders | 1.06 (95% CI 1.00-1.13) |
| F42-49, Other anxiety and stress-related disorders | 0.98 (95% CI 0.95-1.01) |
| F60-62, Personality disorders | 1.00 (95% CI 0.94-1.06) |
| F00-09,35-39,50-59,63-99, Other F-diagnoses | 1.04 (95% CI 1.00-1.09) |
| At least one contact to private psychiatrist | 1.01 (95% CI 0.99-1.04) |
|  |  |
| Severe group overall | 1.00 (95% CI 0.96-1.04) |
| **Severe subgroups** | |
| F20-22 Schizophrenia and psychoses | 0.92 (95% CI 0.87-0.97) |
| F30-31 Bipolar | 1.18 (95% CI 1.07-1.30) |
| F32-34 Unipolar depression with admission | 1.04 (95% CI 0.99-1.10) |
| F60.3 Borderline personality disorder with admission | 0.87 (95% CI 0.74-1.02) |

**List of prior diagnoses for sensitivity analyses**

DQ00-99 Congenital malformations, deformations and chromosomal abnormalities

C00-C96 Malignant neoplasms

D80-D89 Certain disorders involving the immune mechanism (incl. 22q11 deletion syndrome)

Endocrine, nutritional and metabolic diseases (E00-E89)

K. Diseases of the digestive system

K70-77 Diseases of the liver

K80-87 Disorders of gallbladder, biliary tract and pancreas

K90 Intestinal malabsorption (incl. food allergies)

| **eTable 2. Sensitivity analysis excluding children with prior diagnoses. Adjusted incidence rate ratios (IRR) and 95% confidence intervals (95% CI) for the rate of asthma events based on maternal and paternal mental health condition** | | | | |
| --- | --- | --- | --- | --- |
| N = 814,950 |  |  |  |  |
| **Adjusted estimates*** |  |  |  |  |
| **Mother** | Reference group | Minor mental health condition | Moderate mental health condition | Severe mental health condition |
| IRR | 1 | 1.34  (95% CI 1.33-1.35) | 1.32  (95% CI 1.31-1.34) | 1.33  (95% CI 1.28-1.38) |
| **Father** |  |  |  |  |
| IRR | 1 | 1.12  (95% CI 1.10-1.13) | 1.02  (95% CI 1.00-1.04) | 1.02  (95% CI 0.98-1.07) |
| **Adjusted for parental cohabitation status, calendar year, parental age and education, parity, mental health of the other parent and mother and father asthma* | | | | |

| **eTable 3. Number, percentages and adjusted incidence rate ratios (IRR) for the rate of asthma events divided by diagnoses and medication** | | | | | | |
| --- | --- | --- | --- | --- | --- | --- |
| **Asthma medication** |  |  |  |  |  |  |
|  | **Mother** |  |  | **Father** |  |  |
| Mental health condition | N (%) | IRR | 95% CI | N (%) | IRR | 95% CI |
| None | 56476 (8.2) | 1 | | 13551 (1.7) | 1 | |
| Minor | 17274 (10.8) | 1.34 | 1.33 - 1.35 | 2109 (2.1) | 1.12 | 1.07 - 1.17 |
| Moderate | 7812 (11.2) | 1.33 | 1.31 - 1.35 | 1004 (2.3) | 1.07 | 1.00 - 1.14 |
| Severe | 926 (11.5) | 1.37 | 1.33 - 1.42 | 171 (2.4) | 1.03 | 0.89 - 1.18 |
| \| **Asthma hospital diagnosis**  Mental health condition \| \| \|  \|  \|  \|  \|  \| \| --- \| --- \| --- \| --- \| --- \| --- \| --- \| --- \| \| None \| 11366 (1.7) \| 1 \| \|  \| 13551 (1.7) \| 1 \| \| \| Minor \| 3483 (2.2) \| 1.29 \| 1.24 - 1.34 \| \| 2109 (2.1) \| 1.12 \| 1.07 - 1.17 \| \| Moderate \| 1760 (2.5) \| 1.40 \| 1.34 - 1.47 \| \| 1004 (2.3) \| 1.07 \| 1.00 - 1.14 \| \| Severe \| 226 (2.8) \| 1.41 \| 1.25 - 1.60 \| \| 171 (2.4) \| 1.03 \| 0.89 - 1.18 \|   *Adjusted for parental cohabitation status, calendar year, parental age and education, parity, mental health of the other parent and mother and father asthma* | | | | | | |
