# Supplementary figures and images for: Parental mental health, socioeconomic position and the risk of asthma in children—a nationwide Danish register study
Source: Eur J Public Health. 2021 Dec 10;32(1):14–20. doi: 10.1093/eurpub/ckab205 (PMC8807069; doi:10.1093/eurpub/ckab205)

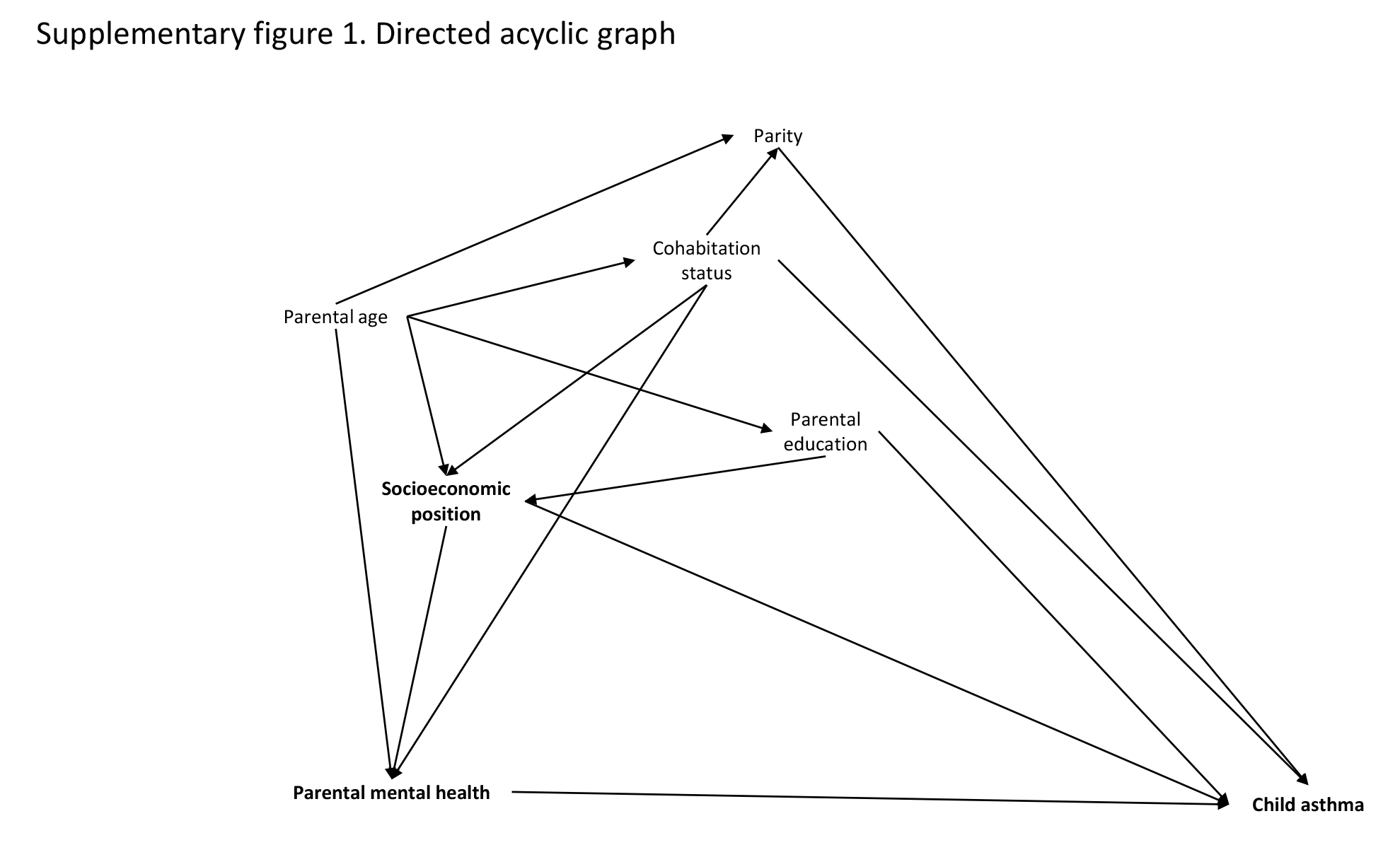

Supplement: ckab205_Supplementary_Data [file ckab205_supplementary_data.zip › ejph-2021-02-om-0244-File004.tif]
